# Elevated Strain Rate Characterization of Compression Molded Direct/In-Line Compounded Carbon Fibre/Polyamide 66 Long Fibre Thermoplastic

**DOI:** 10.3390/ma15217667

**Published:** 2022-10-31

**Authors:** Matthew Bondy, Pouya Mohammadkhani, John Magliaro, William Altenhof

**Affiliations:** Department of Mechanical, Automotive, and Materials Engineering, University of Windsor, Windsor, ON N9B 3P4, Canada

**Keywords:** carbon fibre, thermoplastic resin, impact behaviour, mechanical testing

## Abstract

Compression molded direct compounded carbon fibre D-LFT was evaluated at quasi-static strain rates through uniaxial tension tests (including a specimen size study) and a variation of the ISO 6603-2 puncture test. No significant size effects were observed for the modulus or strength obtained from tensile specimens with four gauge lengths (6.25 mm to 57 mm). Failure strain decreased by 27.5%/29.9%, respectively, across the gauge length range for the 0°/90° directions. Intermediate strain rate (10 s^−1^ to 200 s^−1^) characterization was completed through uniaxial tension tests on a novel apparatus and ISO 6603-2 puncture tests. Intermediate rate tensile tests showed minimal rate sensitivity for the 0°/90° directions. Initial stiffness was 50% higher for ISO 6603-2 impact tests compared to quasi-static tests. Displacement at the onset of fracture was 95% lower for impact tests compared to quasi-static loading. The peak force/displacement at peak force were reduced for impact tests (21% and 20%, respectively) compared to quasi-static testing.

## 1. Introduction

Fibre-reinforced polymers are attractive design options for any engineering applications with economic incentives to minimize mass. This is generally the case for the aerospace industry, but more stringent fuel economy standards and alternative powertrains are among the factors driving the automotive sector to consider structural materials with high specific strength and/or stiffness [1]. Purely in terms of mechanical properties, continuous fibre reinforcement exhibits optimal characteristics in this regard but presents challenges in terms of the manufacturing process and overall cost-effectiveness. Discontinuous fibre reduces challenges with fibre wetout which are generally resolved for continuous fibre with thermoset matrix materials that are more difficult to reuse or recycle, which exacerbates the cost prohibitive disadvantages associated with composite materials [2]. Direct/in-line compounding and compression molding are two processing conditions designed to reduce fibre length attrition to obtain an economically feasible discontinuous fibre material with a recyclable thermoplastic matrix and moderate performance in terms of mechanical properties.

Carbon fibre-reinforced engineering thermoplastics are increasingly considered for automotive applications [3,4,5], however, there are relatively few publications in the open literature, compared to carbon fibre-reinforced thermosets, of studies characterizing their mechanical properties. Several studies focusing on polyamide matrix/LFT composites are summarized in Table 1. One particularly active area of research for carbon fibre-reinforced thermoplastics is surface modification to improve interfacial strength between non-polar carbon fibre and highly polar thermoplastics [2]. A review paper on this topic is included in the table.

High-quality, comprehensive data on direct/in-line compounded carbon fibre-reinforced thermoplastics are particularly scarce. This research is part of an international collaboration between Canadian and German researchers in partnership with Fraunhofer. Table 2 summarizes select publications on fundamental characterization of carbon fibre D-LFT material resulting from this research network. Fundamental characterization of this D-LFT material is presented in [11] including, but not limited to fibre length characterization, fibre orientation distribution characterization by µCT, elastic modulus, and tensile strength. No significant differences between materials of the same fibre content but with different process parameters were observed suggesting an insensitivity to any thermal/mechanical polymer degradation within the range of process parameters considered.

ISO 6603-2 instrumented puncture tests [12] provide evidence of a rate sensitivity in terms of stiffness and deflection at failure. Similar thermoset materials with carbon fibre reinforcement characterized with the same methodology and apparatus showed no sign of inertial effects in terms of stiffness [15]. Carbon fibre alone has been shown to be strain rate insensitive [16] and numerous studies document the strain rate insensitivity of many carbon fibre composites for fibre-dominated properties. A few examples are discussed herein. Modulus, fracture strength, and the failure mode of unidirectional reinforcement carbon fibre-reinforced polymer (epoxy) specimens were independent of strain rate (range between 10^−4^ s^−1^ to 1000 s^−1^) [17]. Melin and Asp [18] assessed the transverse, matrix-dominated mechanical properties of carbon fibre/epoxy composites; the average modulus showed no dependence on strain rate while strain/stress to failure were found to increase slightly with strain rate. Considering only the thermoplastic matrix, polyamide 6 was characterized at strain rates between 10^−2^ and 10^3^ s^−1^ [19]. Flow stress was highly sensitive to strain rate but elastic modulus did not significantly depend on strain rate.

Direct/in-line compounded PA6/CF long fibre thermoplastic was characterized under low-velocity impact consistent with ISO standard 6603-2 [12]; a quasi-static variant of the ISO method was also employed to assess rate sensitivity indirectly. Flow region specimens were notably more brittle considering the force-deflection response at quasi-static loading rates. Puncture energy under low-velocity impacts decreased by 18%, on average, with respect to quasi-static loading. This study prioritized energy absorption and did not compare the force-deflection responses directly between quasi-static loading and low-velocity impact. Concerning the current work, it is important to note that the matrix in [12] was PA6 versus PA66 for the current study. The carbon fibre supplier in [12] was Toho Tenax with sizing for an epoxy matrix while the current study sourced a thermoplastic sized carbon fibre from Zoltek (Panex 35-62).

A lightweight concept for an automotive seatback was evaluated under similar testing conditions to those of the current study (quasi-static and low-velocity puncture consistent with ISO 6603-2) [20]. Digital image correlation (DIC) acquired the three-dimensional displacement fields for a large region spanning the impact surface and regions of the seatback supported by a stiff fixture for both quasi-static and impact loading. Force-deflection responses were compared between quasi-static and low-velocity impact loading with significant differences noted. For small deflections, the force-deflection response was linear for both loading rates, but the stiffness was approximately 550% higher for specimens subjected to low-velocity impact. Digital image correlation showed deflection of the seatback to be more localized within the vicinity of the hemispherical striker for low-velocity impact indicating that even though the impact speed is low [21,22], inertial effects are significant. However, a possible contribution of strain rate sensitivity of the material to the significant increase in stiffness at small deflections could not be conclusively ruled out, and thus this was one of the main driving factors for the current study.

The motivation of the current study was to further develop the observations and findings of a previous study of the ISO 6603-2 puncture test applied to this D-LFT material [12] and impact loading of a D-LFT automotive seatback [20] to advance the understanding of the rate sensitivity observed in these studies. One advantage of the direct compounding process is the customization of the resulting fibre-reinforced thermoplastic through the independent selection of many process parameters including but not limited to thermoplastic resin, carbon fibre content, and tow count/roving number. A robust understanding of the relationship between the material and its constituent components is a necessary preliminary step in optimizing the material for, in this case, applications with impact loading requirements. Therefore, a more fundamental characterization of the strain rate sensitivity of the unreinforced PA66 thermoplastic and the carbon fibre/PA66 composite was carried out under uniaxial tension.

As a precursor to this strain rate dependency study, a uniaxial tension size effect study was completed at a quasi-static strain rate (10^−5^ to 10^−4^ s^−1^). With the assistance of DIC measurements, the seatback study demonstrated the presence of a significant inertial effect. However, it was unknown if this carries over from the seatback resting on a flat plate to the much smaller, clamped specimen of the ISO 6603 method of impact characterization. The inertia effects observed with the automotive seatback may be highly dependent on material properties and specimen geometry. An improved understanding of these dependencies for the ISO 6603 puncture loading methodology is a significant contribution to the engineering community considering the novel and growing field of impact loading of composite materials.

## 2. Materials and Methods

**Figure 1:**
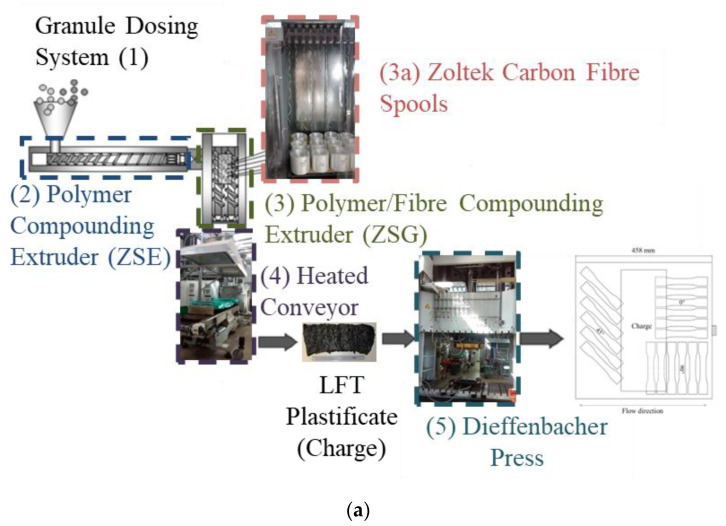

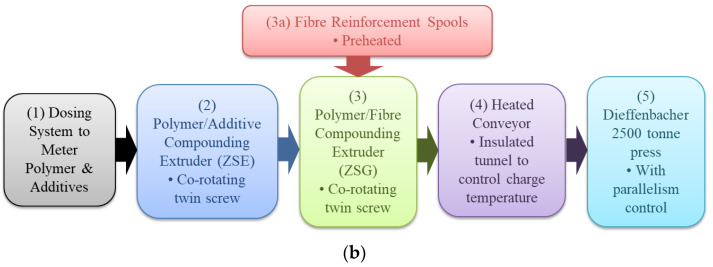
(**a**) Schematic and (**b**) Flowchart of Dieffenbacher DLFT line at the Fraunhofer Innovation Platform for Composites Research at Western University [11].

The direct/inline compounded long fibre thermoplastic (D-LFT) specimens for this study were fabricated on a Dieffenbacher D-LFT manufacturing line at the Fraunhofer Innovation Platform for Composites Research at Western in London, Ontario. A schematic drawing and a flowchart for this manufacturing process are provided in Figure 1a,b, respectively. A Motan granule dosing system (item (1) within Figure 1) feeds a Leistritz ZSE-60HP-28D co-rotating twin screw extruder (2). This extruder introduces molten polymer via a waterfall die (a fully open film die) to a ZSG Leistritz ZSG-75 P-17D (3) co-rotating twin screw extruder (L/D = 17, 75 mm diameter). The carbon fibre roving feed zone (3a) was simplified to remove sharp metallic edges and include the ability to feed the carbon fibre roving directly from center pull bobbins. Charges (approximately 160 mm by 400 mm by 40 mm after cooling and solidifying, note this is not a solid mass of polymer/fibre) exited this extruder, travelled along a heated conveyor (4), and were manually placed in a Dieffenbacher DCP-U 2500/2200 press (5) fitted with a 460 mm by 460 mm flat plaque tool. The temperature set points and measurements for the flat plaque tool are given in Table 3. The press was then closed with a speed of 800 mm/s until a 30 mm offset from fully closed was achieved, followed by 80 mm/s until 5 mm from fully closed, and finally 20 mm/s to fully closed. Once fully closed, the press was held closed for 40 s to allow the polymer to solidify. The polymer was BASF Ultramid^®^ A3W polyamide 66. The carbon fibre was Zoltek Panex 35–62 and the target fibre weight content was 40%. In a previous study of lower fibre content material, fibre lengths ranged from 20 to 5000 µm with an average length of approximately 300 µm [11]. However, many of the longest fibres were entangled and could not be separated for individual length measurement. The average specimen thickness was approximately 3 mm. Specimens were extracted by water jet cutting and dried in a vacuum oven at 100 °C (measured with thermocouples on the specimens) at a vacuum of 70 kPa for one week until a steady state mass reduction of approximately 0.75% was recorded. Specimens were then stored in bags with dried desiccant and mechanical characterization was completed within one week.

**Figure 2:**
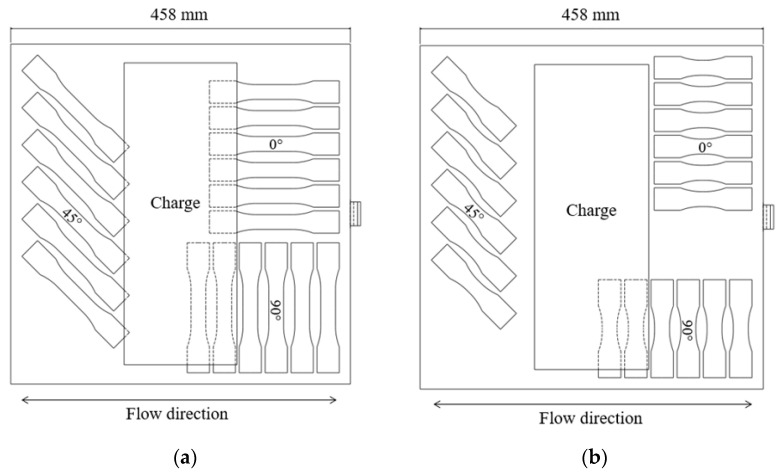
Specimen layouts (**a**) ASTM D638 Type III, (**b**) ASTM D638 Type III with reduced gauge length (6.25 mm shown).

Baseline quasi-static tensile tests at orientations of 0° and 90° with respect to the flow direction (see Figure 2), conducted at strain rates between 10^−5^ s^−1^ and 10^−4^ s^−1^, were completed on an MTS electromechanical load frame with a 25 mm mechanical extensometer and MTS video extensometer employing a 1.3 MP Allied Vision monochrome camera. The crosshead speed was maintained at 1 mm/min, resulting in a strain rate between 10^−5^ s^−1^ and 10^−4^ s^−1^ in the gauge region. The specimen geometry was Type III from standard D638 [23]; Type III specimens possess a 19 mm gauge width and a 57 mm gauge length. The average specimen thickness was 3.06 mm (measurements ranged from approximately 2.9 mm to 3.2 mm). Additional specimens with reduced gauge lengths were evaluated at quasi-static strain rates of 10^−5^ s^−1^ to 10^−4^ s^−1^ (note: average strain rates and coefficients of variation are given in the Results section for each specimen geometry) prior to intermediate strain rate testing to quantify the influence of specimen size on the measured material properties. As the gauge length and width approach the maximum fibre length, reduced gauge dimensions may capture the general response of a material with a modified fibre length distribution compared to that of a significantly larger part. Thus, a specimen size study was performed to determine whether minimizing gauge length is a feasible option to obtain higher strain rates experimentally. SAE standard J2749 [24] provides guidance on specimen geometry from existing ISO and ASTM standards. Grip-to-gauge transitions remained unchanged from the ASTM D638 Type III specimen; however, the gauge length was progressively reduced from 57 mm to 6.25 mm (with intermediate gauge lengths of 25.4 mm and 12.7 mm). All specimen layouts were consistent with only the gauge length reduced, as shown in Figure 2. Quasi-static tensile testing of reduced gauge length specimens inherently limited non-optical strain measurements due to the gauge length of the extensometer. Therefore, the strain was measured optically for all specimens while a mechanical extensometer was only used for validation purposes with specimen geometries where the gauge length was greater than 25 mm.

**Figure 3:**
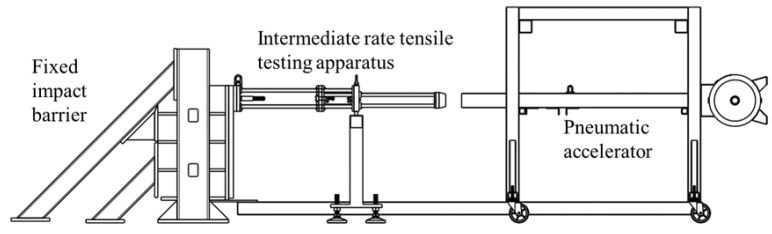
Intermediate strain rate tensile test apparatus integrated with fixed impact barrier and pneumatic accelerator.

**Figure 4:**
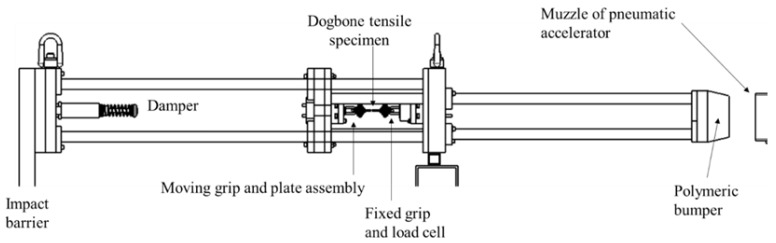
Focused view of the intermediate strain rate tensile apparatus.

Intermediate strain rate (approximately 10 s^−1^ to 200 s^−1^) tensile tests were completed on a custom-designed and engineered apparatus shown in Figure 3 and Figure 4. A 101.6 mm by 101.6 mm hollow structural section (HSS) of steel tubing formed the barrel of the impactor and connected to a pressure vessel which was utilized to accelerate an 8 kg aluminum projectile. This set of components is identified as the pneumatic accelerator in Figure 3. The projectile passed over an aperture in the barrel near the muzzle to disrupt a signal obtained from a laser displacement transducer for triggering a high-speed camera and data acquisition from the load cell. The projectile impacted an assembly (Figure 4) with one unconstrained (translational) degree of freedom (through constraints imposed by seven ceramic linear bearings), connected to a 7000 series aluminum grip assembly which constrained one end of a tensile specimen. The other end of the specimen was held by an identical grip assembly connected to an IEPE load cell (PCB 224C) mounted to a fixed assembly ultimately supported by a large rigid barrier fastened to a concrete floor. The grip design went through multiple iterations to minimize mass (at approximately 50 g per grip, not including the fasteners). For the stiffer and higher strength 0-degree specimens, a small piece of vinyl rubber (approximately 10 mm by 10 mm by 10 mm) with an adhesive pre-applied on one side was attached to the surface of the apparatus impacted by the projectile to reduce vibration in the apparatus resulting from this impact.

The IEPE load cell was powered by a PCB 484B06 signal conditioner (AC coupled), allowing for high-speed data acquisition since the highest possible sampling rate for CompactDAQ modules with IEPE signals as direct inputs are otherwise limited to 102.4 kHz. The force/time data was acquired with a NI 9223 module in a CompactDAQ chassis at 1 MHz. Strain was acquired by post-processing images (128 pixels by 64 pixels) from a Photron Fastcam SA4 high-speed camera with Correlated Solutions ^®^ VIC-2D DIC software. The camera frame rate was 225,000 frames per second triggered with a transistor-to-transistor logic (TTL) signal from a National Instruments 9401 digital input/output module. A custom LabVIEW program was developed to trigger and synchronize data acquisition from the load cell/signal conditioner and trigger the acquisition of high-speed imagery from the camera when the laser displacement transducer registers the passage of the projectile over the aperture in the wall of the barrel of the pneumatic accelerator.

**Figure 5:**
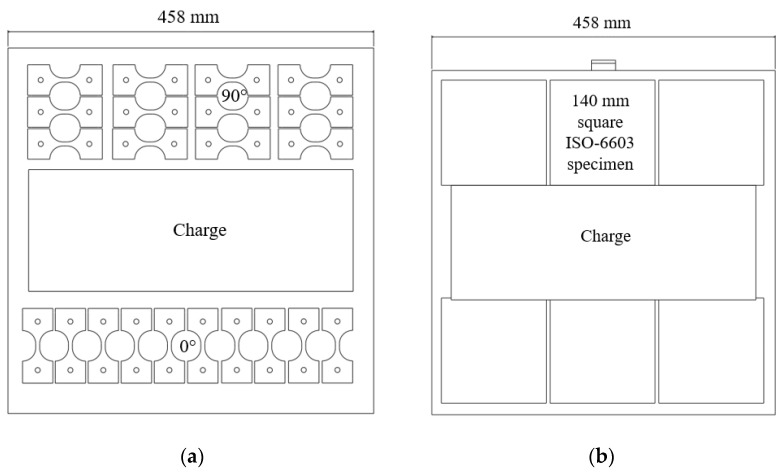
(**a**) Intermediate strain rate specimen layout, (**b**) ISO6603 specimen layout.

The tensile specimen geometry for the intermediate strain rate tests was a modified ASTM D638 Type V geometry (modified to allow a fastener to pass through the gripping regions both to pin the specimen and apply a clamping load through serrated surfaces of the grip). This geometry was utilized in previous studies of similar LFT materials at quasi-static strain rates [11,20]. The gauge length was also reduced from the 9.53 mm length in ASTM D638 to 5 mm to maximize the achievable strain rate. The specimen layout is shown in Figure 5a, note that material availability was limited for these intermediate strain rate tests and the available plaques did not permit every specimen shown to be extracted from each plaque. Multiple plaques were needed to extract the specimens for intermediate strain rate testing.

**Figure 6:**
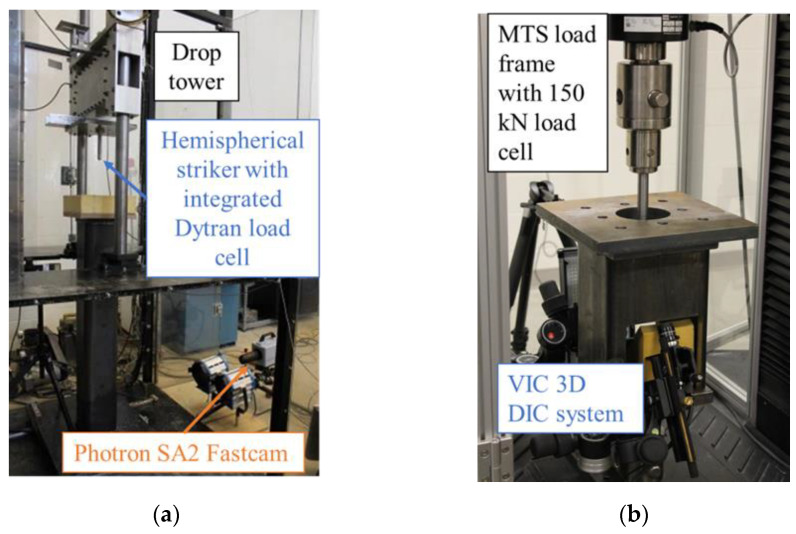
(**a**) Low-velocity ISO 6603-2 apparatus and (**b**) quasi-static puncture apparatus.

Instrumented low-velocity puncture tests were completed consistent with ISO standard 6603-2 on a custom drop tower shown in Figure 6a; per the ISO standard, the impact velocity was 4.4 ± 0.2 m/s. The 140 mm by 140 mm specimen size (specimen layout shown in Figure 5b) was selected following the guideline of the ISO 6603-2 standard for characterization of a brittle material. Force at the 20 mm diameter hemispherical indenter was measured with a Dytran 1050V6 integrated electronics piezo-electric (IEPE) load cell connected to a National Instruments 9250 IEPE capable analog input module with a data acquisition rate of 102.4 kHz. A Photron Fastcam SA4 high-speed camera observed the specimen surface opposite the indenter through a mirror at 72,000 frames per second with a shutter speed of 118,000 s^−1^ at a resolution of 192 pixels by 192 pixels. Camera to load cell data synchronization was implemented with a custom LabView program and a TTL signal from a National Instruments 9401 digital input/output module to the high-speed camera. A dual camera configuration to allow digital image correlation (DIC) was not attempted due to constraints imposed by the drop tower design and fixture.

Quasi-static puncture tests were completed on an electromechanical MTS load frame with a crosshead speed of 0.22 mm/s (0.005% of 4400 mm/s) as shown in Figure 6b. Displacement and strain fields on the specimen surface (100 mm diameter region inside of the clamping ring) opposite the 20 mm hemispherical indenter were acquired with a Correlated Solutions digital image correlation (DIC) system running VIC 3D software. Two Point Grey Grasshopper GRAS-50S5M cameras were triggered with a transistor-to-transistor logic (TTL) signal from the load frame to a NI USB-6221BNC data acquisition device which activated/deactivated the DIC system at the beginning/end of the prescribed crosshead displacement.

## 3. Results and Discussion

### 3.1. Quasi-Static Uniaxial Tensile Tests

Several specimen geometries were utilized to ensure that error was effectively mitigated for measured material properties through modified fibre length distributions. Intermediate to high rate testing inherently required a short gage section both to achieve the strain rate magnitude objective of this study and a state of dynamic stress equilibrium. An SAE draft standard recommends 10 to 15 elastic wave reflection propagations through the gage between the start of loading and yielding [25]. With LFT-D PA66/carbon fibre specimens, the wave speed ($v_{ws}$) in the 90-degree direction is approximately 2200 m/s. For a strain rate of 50 s^−1^ ($\dot{\varepsilon }$), a yield strain of 1% ($\varepsilon_{yield}$), and a gage length of 50 mm ($L_{gauge}$), less than 5 stress wave reflections ($N_{reflect}$) across the gage are expected (from Equation (1)). The only parameter which can be controlled for a desired strain rate and a given material is the gauge length, this necessitates a shorter gauge to achieve higher strain rates. The gauge length should be less than 10 mm to achieve the recommended 15 reflections and a nominal strain rate of 100 s^−1^. Note that the presence of a grip between the load cell and specimen requires that this length be further reduced to achieve the recommended stress wave propagation condition.

(1) $$N_{reflect}=\frac{\varepsilon_{yield}v_{ws}}{2L_{gauge}\dot{\varepsilon }}$$

**Figure 7:**
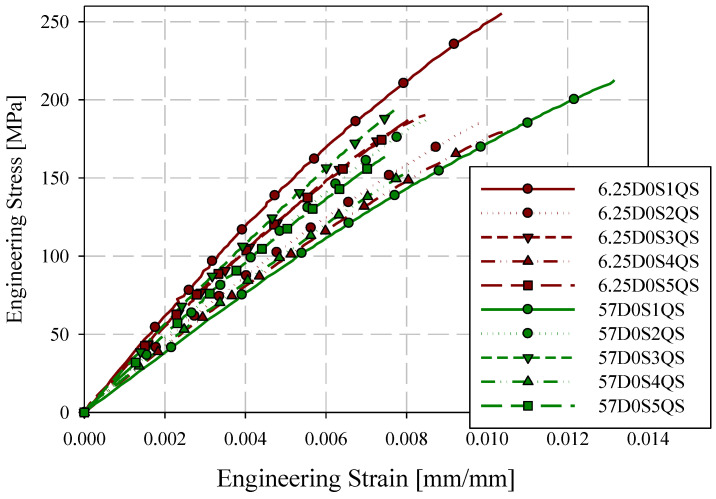
0-degree quasi-static engineering stress-strain responses ASTM D638 type III and reduced gauge length (6.25 mm) specimens.

**Figure 8:**
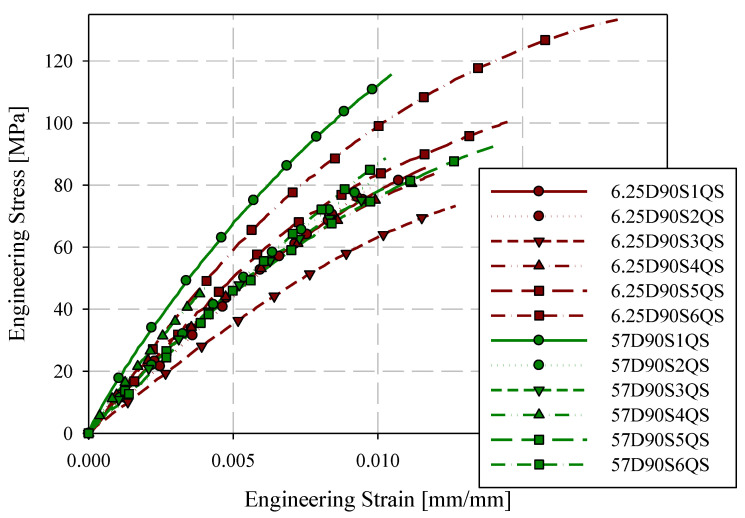
90-degree quasi-static engineering stress-strain responses ASTM D638 type III and reduced gauge length (6.25 mm) specimens.

Quasi-static engineering stress-strain responses for the 0-degree direction are provided in Figure 7 for ASTM D638 Type III and reduced gauge length (6.25 mm) specimens. Specimens are labelled with the format XDYSZQS where X is the gauge length in mm, Y is the orientation with respect to the flow direction (0-degree or 90-degree), Z is the specimen ID (generally starting at 1), and QS indicates a quasi-static loading rate. Stress-strain responses are generally consistent for these two specimen geometries. Quasi-static engineering stress-strain responses for the 90-degree direction are shown in Figure 8. Less consistency between the two specimen geometries was observed with respect to the 0-degree reference direction. However, except for 57D90S1QS, 6.25D90S6QS, and 6.25D90S3QS, the responses were reasonably consistent. The 90-degree specimen layout includes two specimens with proximity to the charge region, which may have less development of flow-induced fibre orientation.

Summaries of quasi-static mechanical properties for all specimens are given in Table 4. For each mean value, the coefficient of variation (expressed in percent) is given in brackets. Considering all mechanical properties summarized in Table 4, no mechanical properties exhibited a high sensitivity to gauge length. Strain to failure with reduced gauge lengths may be slightly reduced with respect to the ASTM D638 Type III standard specimen geometry due to the effect of testing small material volumes, which limits the probability of encountering significant structural defects [13]. However, the change in elastic modulus and tensile strength with respect to specimen size is generally lower than the variation in each mechanical property for any one specimen size, for example, the tensile strength in the 90-degree direction decreased by 0.2% for each additional millimetre of gauge length. Therefore, for the full range of gauge lengths studied (6.25 mm to 57 mm), the average tensile strength decreased by 11.1%. However, the minimum coefficient of variation for 90-degree tensile strength was 13.8% (25 mm gauge length). Failure strain measurements displayed a moderate sensitivity to gauge length. As shown in Table 4, the sensitivity (percent change over the full range of gauge lengths) was generally larger than the coefficient of variation; however, the margin is not intolerably large and the scatter for some individual specimen sizes can still exceed the percent change across the full range of gauge length studied.

### 3.2. Intermediate Strain Rate Uniaxial Tensile Tests

**Figure 9:**
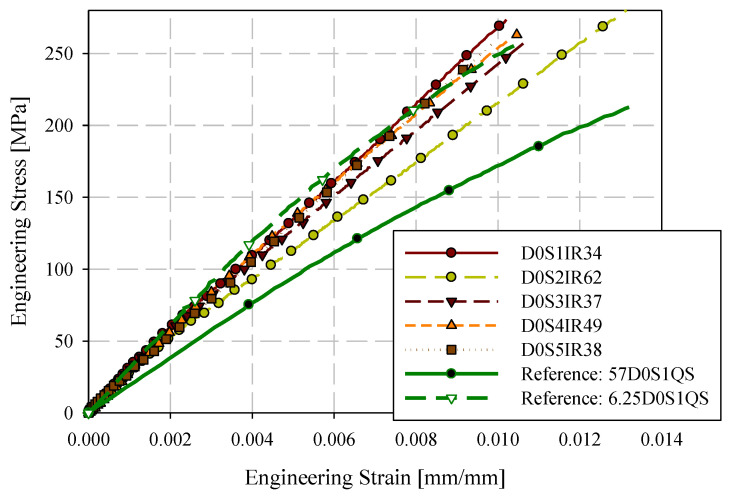
0-degree intermediate strain rate engineering stress-strain responses with upper and lower bound quasi-static reference responses.

Uniaxial tension test stress-strain results from the intermediate strain rate tension testing apparatus, obtained for the 0-degree direction, are shown in Figure 9. Specimens were labelled with the format DXSYIRZ where X is the orientation of the specimen with respect to the flow direction (0° or 90°), Y is the specimen ID (generally starting at S1), IR indicates an intermediate strain rate (10 s^−1^ to 200 s^−1^), and Z is the strain rate (s^−1^). The intermediate strain rate results skew towards the upper limit observed for the quasi-static response, also presented in Figure 9. The stress-strain responses are significantly more linear than the complementary quasi-static results which may be indicative of minimal positive strain rate sensitivity. The stress-time (obtained from load-time with the initial specimen gauge cross-section) and strain-time (from DIC) were cross-plotted by fitting a small number of spline segments (approximately 3) to the strain-time data and increasing the time domain resolution to equal that of the load-time data. The load data was not manipulated in any way; no filters or smoothing were applied when post-processing the data.

**Figure 10:**
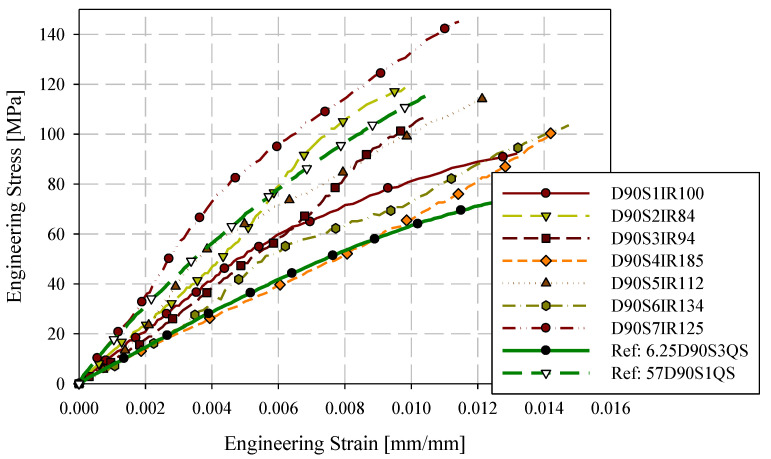
90-degree intermediate strain rate engineering stress-strain responses with upper and lower bound quasi-static reference responses.

Intermediate strain rate results for 90-degree specimens are shown in Figure 10. There is a wide corridor for these results but this is consistent with previous studies [11,20]. The 90-degree direction is perpendicular to the flow direction towards which the fibre will orientate. However, depending upon the specific position of each specimen in the plaque and randomness in the fibre orientation of the charge, the small fraction of fibres reinforcing the 90-degree direction will vary significantly. These tests did not employ the vinyl rubber impact attenuator which, in pilot testing, did provide a level of mechanical filtering of the impact load. Only the matrix is expected to influence any observed strain rate sensitivity due to the expected lack of rate sensitivity associated with the carbon fibre reinforcement [16]. Therefore, it may be that any positive strain rate sensitivity of the matrix in the 0-degree direction only counteracts the stiffness reduction observed at quasi-static strain rates but for the 90-degree direction, which will be far more sensitive to matrix properties, any positive strain rate sensitivity of the matrix is observed as a slight stiffening. Since the vinyl rubber attenuator was not used, the strain rates are higher than the 0-degree intermediate strain rate tests. This is another factor to be considered in understanding the 90-degree responses compared to the 0-degree responses in terms of stiffness.

### 3.3. Quasi-Static Puncture Tests

**Figure 11:**
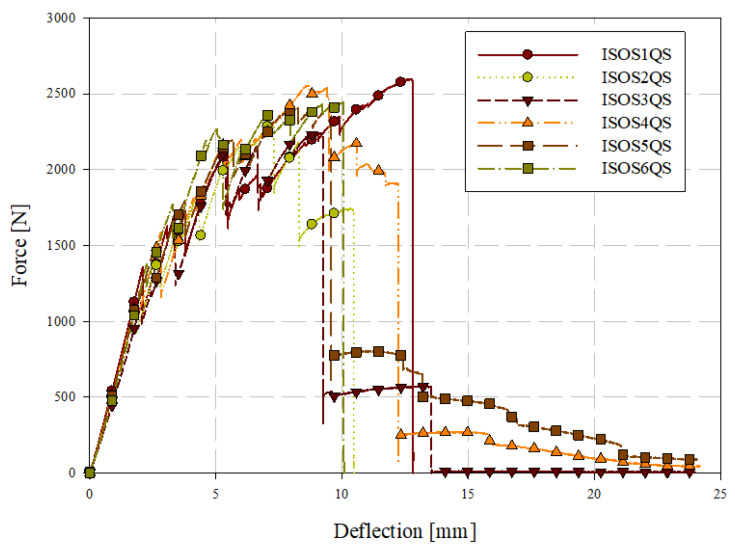
Quasi-static puncture force-deflection responses.

**Figure 12:**
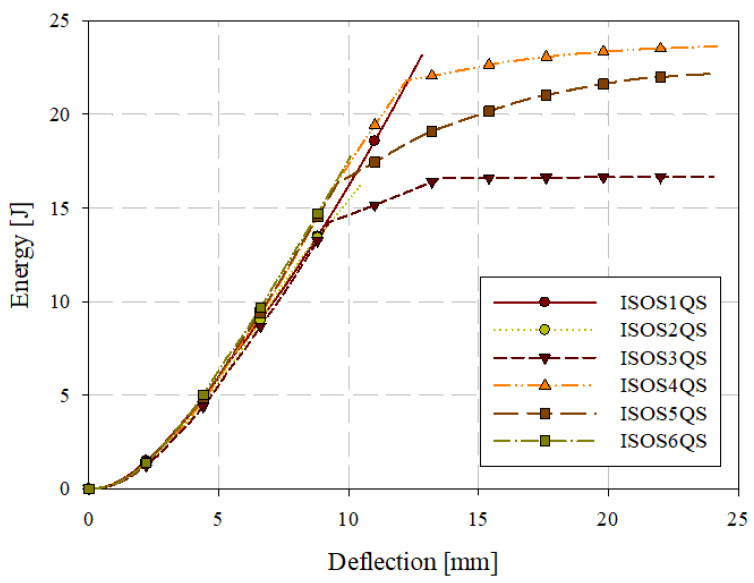
Quasi-static puncture energy-deflection responses.

Quasi-static puncture testing was conducted consistent with ISO standard 6603-2, but with the velocity reduced to 0.22 mm/s or 0.005% of the ISO 6630 impact speed of 4.4 m/s. Specimens were labelled with the format ISOSXQS where X is the specimen ID (starting at 1). Force-deflection data from this series of tests are shown in Figure 11. Individual quasi-static and low-velocity impact force-deflection responses are presented in Section 3.5. This drastic reduction in loading rate allowed for the use of high-resolution cameras with the digital image correlation (DIC) system while acquiring high-resolution data in the deflection domain. Table 3 presents the consistency in general force-deflection responses in terms of initial stiffness and peak load and the corresponding deflection at peak load. Energy-deflection responses are shown in Figure 12. Force-deflection and energy-deflection responses are generally consistent for all six quasi-static specimens until the onset of catastrophic failure. Energy-deflection responses up to approximately 10 mm of deflection were very consistent. Force-deflection responses, even at deflections as low as approximately 2.5 mm, exhibit variation associated with the initiation and propagation of fracture.

A tabular summary of the results of quasi-static puncture testing is presented in Table 5. The elastic stiffness was computed by fitting a straight line to the force-deflection response from 0 mm of deflection up to the maximum deflection at which a linear fit was appropriate. For quasi-static loading, this was the deflection at which fracture initiated, which was approximately 2 mm. Energy is the total energy absorbed up to the maximum displacement in Figure 12 for each specimen. The deflection and first principal strain at the onset of fracture were identified from DIC images.

**Figure 13:**
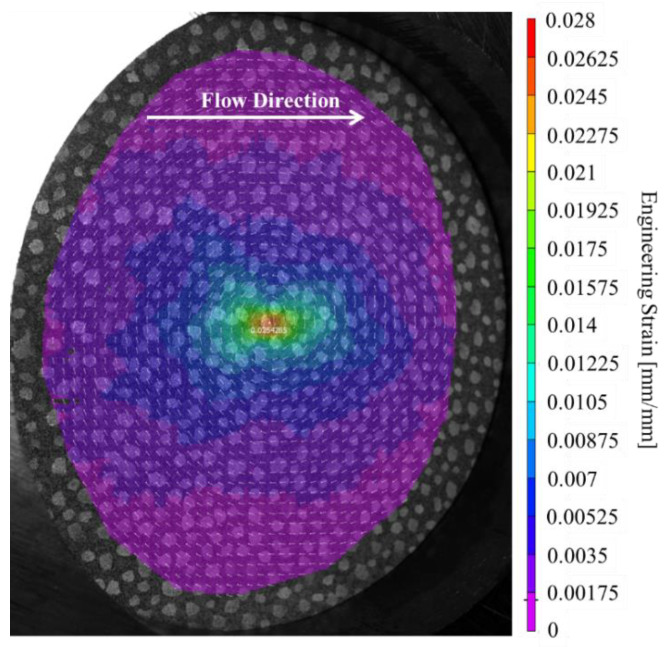
1st principal (engineering) strain from DIC for quasi-static specimen #1.

A single DIC image of the contours of the first principal engineering strain immediately prior to the onset of fracture for quasi-static puncture of specimen ISOS1QS is shown in Figure 13. The maximum principal engineering strain under loading with a hemispherical indenter was higher than observed for uniaxial tensile tests, being an average of 3.6% for quasi-static puncture versus 1.1% for uniaxial tension tests. The specimen underwent biaxial tension and bending when subjected to puncture loading at the midspan. However, another key difference between these tests is the source of this strain data. The engineering strain at failure for quasi-static puncture is a localized measurement of peak strain while the strain at failure for uniaxial tension is distributed over the gauge region within the contact areas of the clips of the extensometer or between the two points tracked by the video extensometer. To better understand the difference between the maximum local engineering strain and the average engineering strain over a large region, two-dimensional DIC was employed to obtain localized maximum strain for select tensile tests. Tensile specimen failure was observed to occur outside the small region of the gauge in most tensile tests, for which images were collected for DIC. For specimens where failure occurred in the region analyzed with DIC, the maximum strain was similar to the extensometer value being within 0.1% strain.

### 3.4. Low-Velocity ISO 6603-2 Puncture Tests

**Figure 14:**
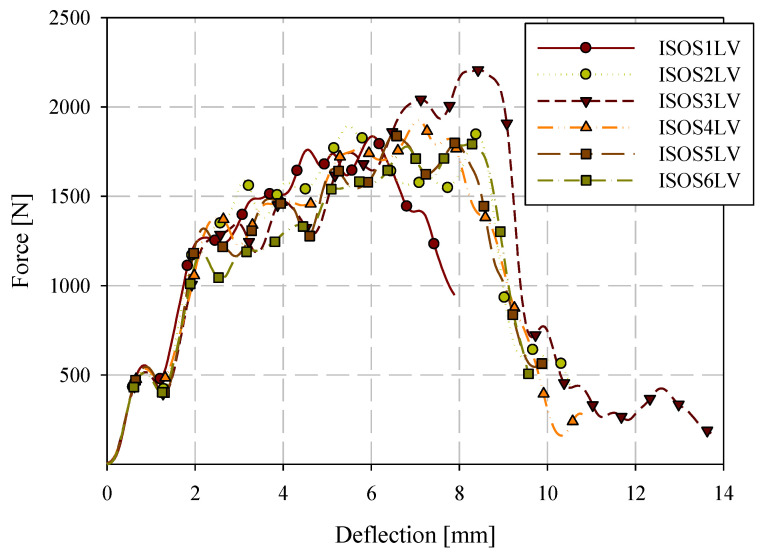
Filtered low-velocity impact puncture force-deflection responses.

**Figure 15:**
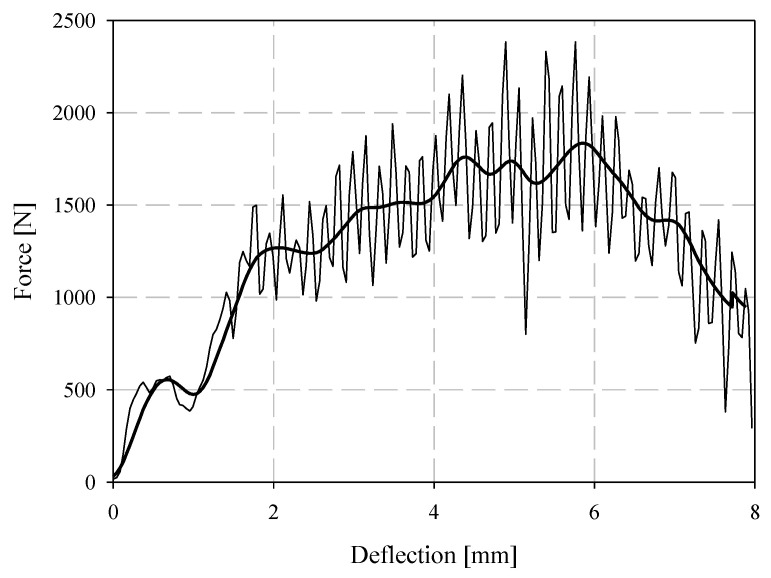
Filtered and unfiltered force-deflection response for low-velocity impact specimen ISOS1LV.

**Figure 16:**
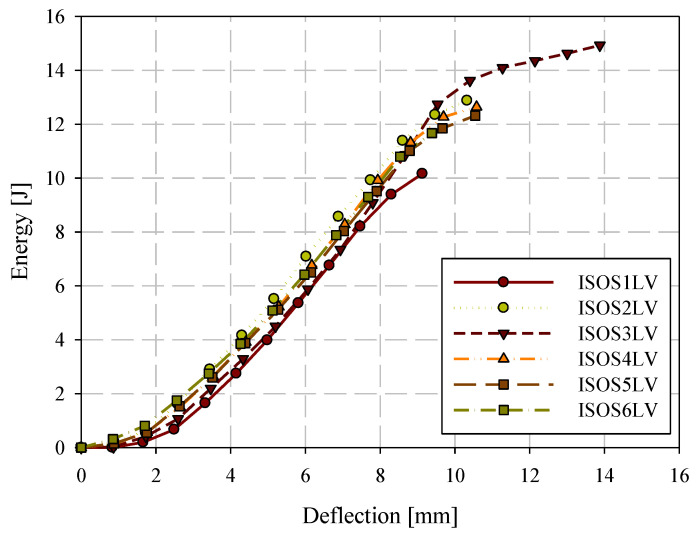
Low-velocity impact puncture energy-deflection responses.

Low-velocity (LV) ISO 6603-2 puncture specimens were labelled with the format ISOSXLV where X is the specimen ID (starting at 1). Filtered force-deflection responses for low-velocity puncture testing, completed in accordance with the ISO 6603-2 standard, are shown in Figure 14 for all six specimens tested and a single force-deflection response is shown for specimen ISOS1LV in Figure 15 (both filtered and unfiltered). A 2-pole Butterworth filter with forward and reverse passes (resulting in a 4-pole filter) with a channel frequency class (CFC) of 1000 was applied. This is the highest frequency cut-off low-pass filter specification in SAE J211 [26]. Compared to the quasi-static results, the change in force associated with the initiation and propagation of fractures were not captured well in either the filtered or unfiltered responses. Energy-deflection responses are shown in Figure 16. In terms of energy-deflection, all six specimens show very similar responses with only some variation in the displacement to failure and the peak energy dissipated through the puncture testing.

A tabular summary of the results of low-velocity puncture is presented in Table 6. The elastic stiffness was computed by fitting a straight line to the force-deflection response from 0 mm of deflection up to a deflection where a linear fit was appropriate; the deflection domain for this linear fit was approximately 0.5 mm. The maximum force and coinciding deflection were based on filtered data. Energy represents the total energy absorbed up to the maximum displacement in Figure 16 for each specimen. The deflection at the onset of fracture was identified from high-speed imagery; best attempts were made to assess given the camera acquisition rate and resolution.

### 3.5. ISO 6603 Puncture Test—Comparison of Quasi-Static and Low-Velocity Responses

**Figure 17:**
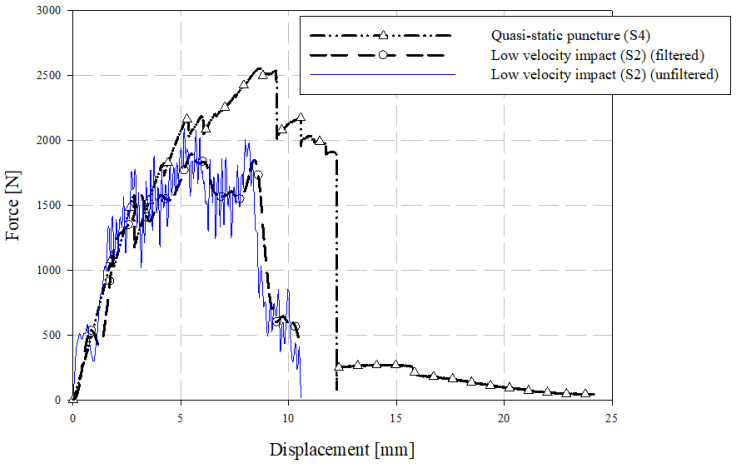
Comparison of quasi-static and low-velocity puncture force-deflection responses.

**Figure 18:**
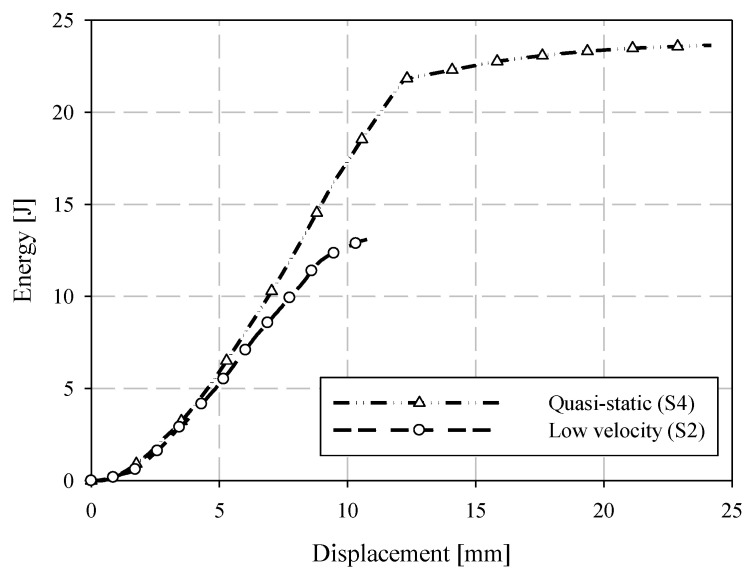
Comparison of quasi-static and low-velocity puncture energy-deflection responses.

Representative samples from quasi-static and low-velocity puncture tests are compared in terms of force-deflection responses in Figure 17 and energy-deflection responses in Figure 18. At small deflections, the force-deflection responses are similar, though not identical, at the two loading rates considered. In previous ISO 6603-2 puncture tests [12] and similar tests of automotive seating components loaded with the same hemispherical indenter [20], polyamide/carbon fibre LFT exhibited significant rate effects (due to inertial and/or strain rate phenomena) at small deflections. The material was not identical, but in the case of the automotive seatback, the matrix and fibre were from the same supplier and the compounding process was identical. Flow-induced fibre orientation in the mold is the only differentiating factor between the material in the current study and the automotive seatback study. This indicates geometric and material dependency on rate effects, and that there is little to no strain rate effect in terms of the observed elastic modulus. In terms of rate effects, this earlier onset of failure is significant. This earliest onset of failure has a significant effect on damage propagation and the ultimate failure and energy absorption due to the brittle nature of carbon fibre-reinforced materials.

**Figure 19:**
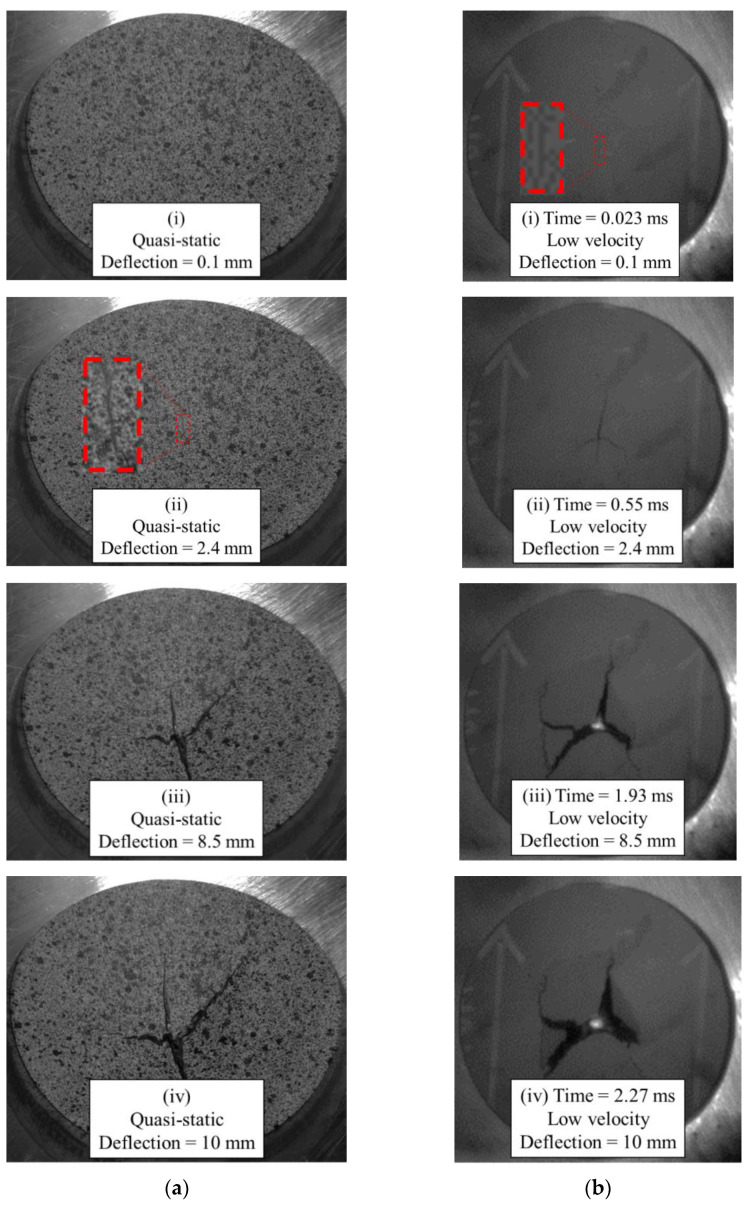
Images of specimen face opposite indenter: (**a**) quasi-static, (**b**) low-velocity impact.

Imagery of the specimen faces opposite to the indenter is compared between loading rates in Figure 19. Note that the quasi-static images are not as perpendicular to the face of the undeformed specimen since the images are acquired from one of the two cameras of a DIC system with an angle between the cameras sufficient to resolve out-of-plane displacements. A crack is visible at approximately 2 mm of quasi-static crosshead displacement. Under low-velocity impact loading, a crack appears at a dramatically lower magnitude of deflection, that being approximately 0.1 mm. This observation may be a further indication of the dynamic nature of the ISO 6603-2 puncture test, particularly, there may be localized deformations with much higher strains for equal striker displacements between quasi-static and low-velocity tests. The flow direction is shown on the impact test specimens with a large arrow and also applies to the neighboring quasi-static specimen image. In both cases, the crack initially forms parallel to the flow direction (approximately parallel to the nominal fibre direction). At displacements in the range of 8.5 mm to 10 mm, circumferential cracks are generally present under low-velocity loading that are not visible in quasi-static tests. These circumferential cracks cause large segments of the specimen to break off which drastically reduces the load-carrying capacity of the specimen. Comparing data within Table 3 and Table 4, the initial stiffness was approximately 50% higher for low-velocity puncture testing compared to the complementary quasi-static value. This indication of inertial effects presents challenges for understanding material behaviour from this puncture test.

### 3.6. Quasi-Static and Low-Velocity Impact Loading of an LFT-D Automotive Seatback

**Figure 20:**
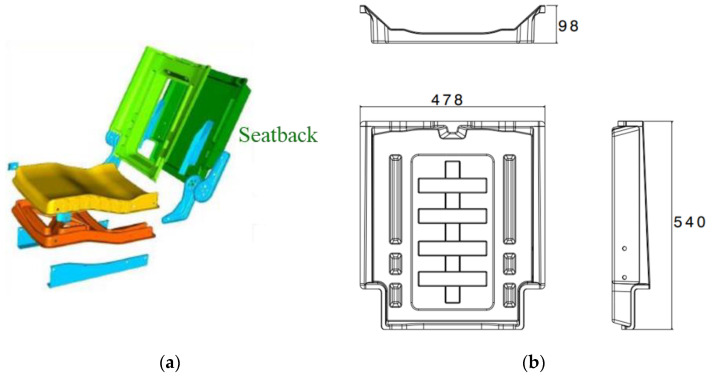
LFT-D Automotive Seatback: (**a**) as part of the assembly, (**b**) overall dimensions [mm].

**Figure 21:**
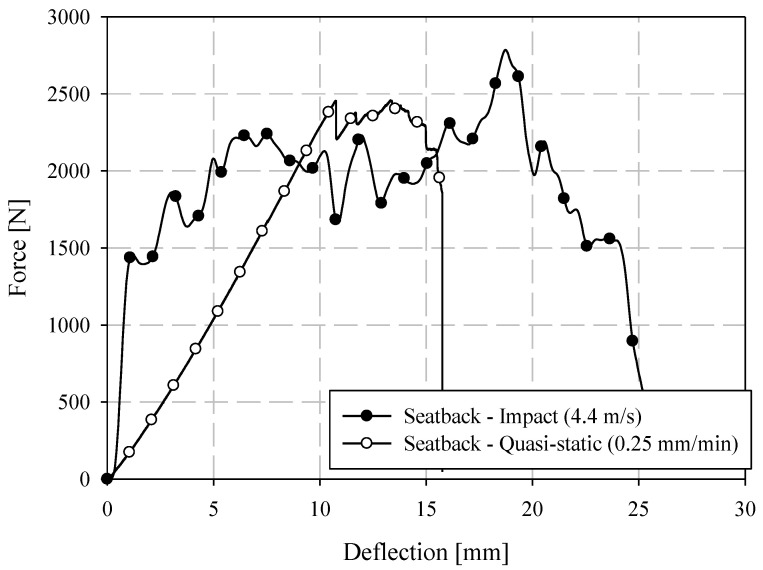
Comparison of quasi-static and low-velocity impact test force-deflection responses of carbon fibre LFT seatback components.

**Figure 22:**
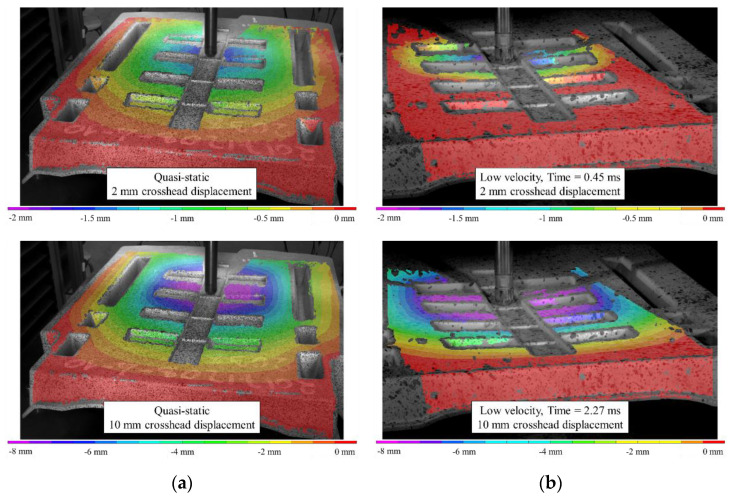
(**a**) Quasi-static and (**b**) low velocity impact loading comparison for longitudinal charge seatbacks: z-deflection contours.

A previously published study, summarized in the introduction, documented rate sensitivity of a PA66/carbon fibre LFT-D automotive seatback subjected to similar loading with a hemispherical indenter [20]. The seatback, shown in Figure 20 (the seatback is dark green), was comprised of the same fibre and matrix material in unchanged proportions processed with the same extruders as the tensile and ISO 6603-2 specimens previously discussed. However, since the fibre orientation is the result of transient material flow in the mold, due to the geometry of the seatback the fibre orientation is therefore expected to differ significantly. As previously noted, the puncture tests strictly following the ISO 6603-2 standard showed an average initial stiffness 50% greater than quasi-static tests. Previous work on lower carbon fibre content specimens similarly showed a significant difference between quasi-static and low-velocity impact loading following the ISO 6603-2 standard [12]. This is much lower than the increased stiffness with an increased loading rate observed with the automotive seatback at 550% (Figure 21). Comparing the displacement fields from DIC (Figure 22) in conjunction with the force-deflection responses shows that at displacements less than 10 mm there is a large difference in the force for a given deflection. Correspondingly, the displacement fields show that deformation is much more localized to the region around the hemispherical indenter under impact providing strong evidence of an inertia effect. When the displacement reaches approximately 10 mm, the forces for quasi-static and low-velocity impact loading have nearly equalized. DIC shows that at this level of deflection there is no longer significant localization of deflection around the indenter for low-velocity impact.

Considering this prior work on similar carbon fibre LFT materials and the results of the current study, the ISO puncture test should be employed with caution for brittle materials, particularly where quantifying energy absorption is a primary objective. The ISO 6603-2 standard specifically includes guidelines for testing such materials but does not include any discussion on the challenges and limitations of testing such materials for energy absorption. As computed in ISO 6603-2 from the indenter force and displacement, energy absorption quantifies a notably different energy transfer process for ductile materials versus brittle materials. In a ductile material, the work done by the indenter has a significant component dissipated through plastic deformation. Additionally, for a ductile material the specimen is essentially stationary shortly after the indenter punctures the specimen, therefore, the specimen has no significant kinetic energy in this final state. With a brittle material, shortly after puncture there will likely be large fragments of the specimen which are moving with velocities that are not negligible and therefore possess significant kinetic energies. The entire specimen will also vibrate after any catastrophic failure as the elastic internal energy is suddenly converted to kinetic energy. Compared to a ductile material, much less energy is absorbed by plastic deformation while more energy is absorbed through crack propagation in the brittle specimen. For a composite material consisting of a large strain to failure matrix and relatively brittle reinforcing fibre (as is the case here), the volume fraction of the matrix undergoing large plastic deformations will also be significantly lower than for an unreinforced polymer specimen. Under impact loading, energy absorptions of multiple materials with similar constituents (i.e., different fibre contents) may have different contributions from kinetic energy. Any such results obtained with the standard ISO 6603-2 specimen are likely not applicable to offer guidance in the design of complex geometries, e.g., commercial products.

## 4. Conclusions

The mechanical response of compression molded direct/in-line compounded carbon fibre LFT was evaluated at quasi-static strain rates through uniaxial tension tests and puncture testing as well as at intermediate strain rates (10–100 s^−1^) through uniaxial tension testing with a novel apparatus and ISO 6603-2 instrumented puncture tests. The quasi-static uniaxial tension tests included a study of specimen size effects. The key findings of this investigation are as follows:

1. Quasi-static uniaxial tension tests with 4 specimen gauge lengths ranging from 6.25 mm to 57 mm did not exhibit evidence of a significant size effect for elastic modulus or tensile strength. Failure strain was moderately sensitive to specimen gauge length. For the 0-degree direction, the failure strain decreased by 27.5% across the full range of gauge length studied (coefficient of variation for 0-degree specimens: 19.2%). For the 90-degree direction, the failure strain decreased by 29.9% across the full range of gauge length studied (coefficient of variation for 90-degree specimens: 22.6%).
2. Intermediate strain rate tensile tests showed little to no strain rate sensitivity for both the 0-degree and 90-degree directions. This is expected since carbon fibre has little to no rate sensitivity and PA66 is not highly strain rate sensitive.
3. Compared to quasi-static tensile tests, the 0-degree intermediate strain rate tests possess a more linear response compared to the decreasing stiffness (with increasing strain) of the quasi-static tests. This may be indicative of a slightly positive strain rate sensitivity of the PA66 matrix since the strain rate is ramping up throughout the test, particularly for 0-degree tests where an impact attenuator was utilized to reduce the vibration of the system.
4. The 90-degree intermediate strain rate tests were characterized by a nonlinear response. This was likely caused by positive strain rate sensitivity of the PA66 matrix, to which the 90-degree tests are more sensitive. However, another factor to consider is that the 90-degree tests did not employ a vinyl rubber impact attenuator (which was present for the 0-degree tests), and thus the strain rate ramped up more rapidly and more vibration was present in the system for the novel apparatus used within this investigation.
5. Low-velocity ISO 6603-2 instrumented puncture tests were completed. A quasi-static variation of this material characterization methodology was also conducted on a load frame. The initial stiffness was approximately 50% higher for low-velocity impact tests compared to the quasi-static tests and the displacement at the onset of fracture was much lower, approximately 95% lower, for low-velocity impact tests. Correspondingly, the displacement at the peak force and the peak force were reduced for low-velocity impact tests, 20% and 21%, respectively.
6. The ISO 6603-2 low-velocity impact test methodology should be employed with caution for brittle materials since the kinetic energy of the specimen (and fragments thereof) as the specimen fails may be significant relative to the internal energy. Significant dynamic effects were present in the considered testing, additionally, this dynamic effect was highly sensitive to material properties and specimen geometry.
7. A seatback comprised of a consistent material to the current study loaded in a similar condition had a 550% increase in initial stiffness under low-velocity impact conditions compared to quasi-static tests. DIC analysis revealed the deformation to be more localized to the indenter for low-velocity impact tests at small displacements. This dynamic effect was observed to be significant well before failure of the specimen.

## Figures and Tables

**Table 1:** Carbon-reinforced thermoplastic literature review.

Material	Summary	Reference
PA6/CF (2–20%)	Modulus and tensile strength increased with fibre content. Failure strain decreased. Glass transition temperature did not change significantly with fibre content.	[6]
PA66/CF (20–30%)	Short fibre and LFT materials compared with improved tensile modulus and strength with increased fibre length.	[7]
PA6/clay/CF (10–30%)	Nanoscale clay increased tensile/flexural strength and modulus without reducing impact strength	[8]
PA6/PP Blends 20% CF	PA6/PP blend to reduce the sensitivity of PA6 tensile strength and elastic modulus to moisture content.	[9]
PP/CF (LFT)	Increasing tensile/flexural and impact strength up to 25% fibre. Hypothesized that at higher fibre contents increasing void content degrades properties.	[10]
Various polymers/CF	Review paper on surface modification of carbon fibre for polar thermoplastic matrix materials	[2]

**Table 2:** Carbon-reinforced long fibre thermoplastic research by the International Composites Research Group.

Material	Summary	Reference
PA6/CF (9–25%)	Fundamental mechanical characterization including elastic and flexural moduli, tensile strength, and strain to failure. µCT characterization of fibre orientation. Fibre length characterization.	[11]
PA6/CF (9–25%)	ISO 6603-2 instrumented puncture impact testing.	[12]
PA66/CF (40%)	Tension-tension (R = 0.1) stress-life fatigue characterization and SEM studies of fracture surfaces.	[13]
PA66/GF PA66/CF	TGA, DSC, and GPC characterization.	[14]

**Table 3:** Tool temperature set points and measurements.

Notes	Cavity	Core
Set point at beginning of the trial	135 °C	140 °C
Initial surface temperature readings	110 °C	112 °C
Surface temperature after 25 charges	119 °C	127 °C
Surface temperature after 50 charges	120 °C	126 °C
Surface temperature after 75 charges	120 °C	128 °C
Set point after lunch break	145 °C	150 °C
Initial surface temperature readings	124 °C	126 °C
Surface temperature after end of trials	130 °C	136 °C

**Table 4:** Summary of quasi-static uniaxial tensile test results (coefficient of variation in parentheses).

Specimen Group	Strain Rate [s^−1^]	Elastic Modulus [GPa]	Tensile Strength [MPa]	Engineering Strain at Failure [%]	Poisson’s Ratio [υ]
6.25D0	4.9 × 10^−5^ (15.1%)	24.6 (15.0%)	201.2 (15.8%)	1.34 (21.2%)	0.358 (10.7%)
12.7D0	6.6 × 10^−5^ (8.5%)	24.0 (8.5%)	206.8 (6.5%)	1.06 (15.3%)	0.426 (15.4%)
25D0	6.2 × 10^−5^ (20.8%)	20.6 (5.7%)	182.9 (9.4%)	1.01 (14.7%)	0.506 (17.8%)
57D0	5.8 × 10^−5^ (20.2%)	23.1 (13.1%)	186.3 (12.1%)	0.90 (25.6%)	0.401 (24.7%)
0° Sensitivity to Gauge Length	2.3%	−5.8%	−9.6%	−27.5%	3.5%
6.25D90	1.6 × 10^−4^ (18.5%)	9.45 (18.4%)	93.3 (23.4%)	1.33 (23.4%)	0.155 (33.9%)
12.7D90	1.3 × 10^−4^ (16.2%)	9.42 (17.0%)	96.9 (13.9%)	1.34 (21.2%)	0.206 (37.6%)
25D90	1.3 × 10^−4^ (16.6%)	8.34 (8.3%)	89.4 (13.8%)	1.18 (14.7%)	0.207 (34.8%)
57D90	1.0 × 10^−4^ (11.5%)	10.3 (18.2%)	85.1 (25.9%)	0.99 (31.2%)	0.190 (30.3%)
90° Sensitivity to Gauge Length	−35.9%	10%	−11.1%	−29.9%	8.1%

**Table 5:** Summary of quasi-static puncture mechanical responses.

Specimen Identifier	Elastic Stiffness [N/mm]	Maximum Force [N]	Deflection at Maximum Force [mm]	Absorbed Energy [J]	Deflection at Onset of Fracture [mm]	Principal Strain at Onset of Fracture [%]
ISOS1QS	650	2596	12.7	23.1	2.2	2.5
ISOS2QS	609	2297	7.2	16.2	2.3	2.7
ISOS3QS	550	2250	8.9	16.7	2.1	3.3
ISOS4QS	620	2554	8.7	23.6	2.4	5.7
ISOS5QS	618	2415	9.5	22.2	2.2	3.8
ISOS6QS	605	2444	10.0	17.7	2.4	3.4
Average	609 N/mm	2426 N	9.5 mm	19.9 J	2.3 mm	3.6%
Coefficient of variation	5.4%	5.6%	19.3%	17.1%	5.3%	32.2%

**Table 6:** Summary of low-velocity puncture mechanical responses.

Specimen Identifier	Elastic Stiffness [N/mm]	Maximum Force [N] (Filtered)	Deflection at Maximum Force [mm]	Absorbed Energy [J]	Deflection Fracture Onset [mm]
ISOS1LV	983	1836	7.3	10.2	<0.1
ISOS2LV	963	1895	6.0	13.2	<0.1
ISOS3LV	958	2207	9.3	15.0	0.1
ISOS4LV	967	1927	7.7	12.8	<0.1
ISOS5LV	1016	1842	7.1	12.3	<0.1
ISOS6LV	989	1793	8.4	11.8	<0.1
Average	979 N/mm	1917 N	7.6 mm	12.6 J	<0.1 mm
Coefficient of variation	2.2%	7.8%	14.9%	12.7%	-

## Data Availability

The data contained within this manuscript is part of an ongoing study and at this time data sharing is not applicable.
